# Classification of coal bursting liability of some chinese coals using machine learning methods

**DOI:** 10.1038/s41598-024-61801-0

**Published:** 2024-06-18

**Authors:** Chao Wang, Yv Liu, Yuefeng Li, Xiaofei Liu, Qiwei Wang

**Affiliations:** 1 Faculty of Land Resources Engineering, Kunming University of Science and Technology, Kunming, 650093 China; 2 State Key Laboratory of Hydraulics and Mountain River Engineering, College of Water Resource and Hydropower, Sichuan University, Chengdu, 610065 China; 3 School of Safety Engineering, China University of Mining and Technology, Xuzhou, 221116 China

**Keywords:** Bursting liability classification, Machine learning, Performance evaluation, Model optimization, Engineering application, Civil engineering, Computer science

## Abstract

The classification of coal bursting liability (CBL) is essential for the mitigation and management of coal bursts in mining operations. This study establishes an index system for CBL classification, incorporating dynamic fracture duration (*DT*), elastic strain energy index (*W*_ET_), bursting energy index (*K*_E_), and uniaxial compressive strength (*R*_C_). Utilizing a dataset comprising 127 CBL measurement groups, the impacts of various optimization algorithms were assessed, and two prominent machine learning techniques, namely the back propagation neural network (BPNN) and the support vector machine (SVM), were employed to develop twelve distinct models. The models’ efficacy was evaluated based on accuracy, F1-score, Kappa coefficient, and sensitivity analysis. Among these, the Levenberg–Marquardt back propagation neural network (LM-BPNN) model was identified as superior, achieving an accuracy of 96.85%, F1-score of 0.9113, and Kappa coefficient of 0.9417. Further validation in Wudong Coal Mine and Yvwu Coal Industry confirmed the model, achieving 100% accuracy. These findings underscore the LM-BPNN model’s potential as a viable tool for enhancing coal burst prevention strategies in coal mining sectors.

## Introduction

Coal^1^ serves as a fundamental source of energy and a crucial industrial raw material essential for the advancement of human civilization. Within the realm of mining engineering, ensuring the safe extraction of coal stands as a paramount objective. Moreover, proactive measures^2,3^ in early detection and scientific prevention of disasters arising from coal mining operations and engineering constructions form the cornerstone of coal mining practices.

Coal mines are prone to various disasters^4–6^. As a dynamic disaster severely threatening to the safety production of coal mines^7–10^, coal burst often causes mine tunnel damage, casualties, and economic losses. Coal burst is affected by many factors, such as components of coal-rock mass, energy, and mining disturbance. Based on different properties of coal, Bieniawski et al.^11,12^ proposed the bursting liability theory, who pointed that bursting liability is an inherent property of coal-rock mass for burst failure^13^. The “Rules for Prevention and Control of Coal bursts in Coal Mines,” issued by the National Coal Mine Safety Supervision Administration of China, stipulates that coal seams that have experienced coal burst within the mine field, or coal seams that have been identified as having coal burst tendency (or rock strata of roof and floor) and are assessed as having coal burst risk are coal burst coal seams. The mine with coal burst coal seam is a coal burst mine. Coal bursting liability^14^, serving as an intrinsic factor and essential condition for the occurrence of coal bursts, has emerged as a key basis for evaluating and predicting coal burst hazards in coal mines. As an internal cause and necessary condition of coal bursts, CBL of different coals is significant difference.

Therefore, judging the class of CBL accurately is essential for the prevention and control of coal bursts. Mathematical and statistical methods have been widely adopted in classification and evaluation problems, as well as in CBL classification. Guided by theory of unascertained measure model, Xu et al.^15^ established a comprehensive model for CBL classification by applying confidence degree to sorting and calculating index weight with information entropy. According to the entropy weight-ideal point method, Wang et al.^16^ developed a CBL classification model, and achieved good engineering effect. Jia et al.^17^ introduced the attribute mathematics theory, determined the weight of each index with the similar number and similar weight method, identified the attribute by the confidence criterion, and established an attribute mathematical model for CBL classification. Guo^18^ determined the grey distance correlation degree of the evaluation object through the grey correlation analysis, constructed the grey identification model for CBL classification according to the minimum membership degree principle. Wang et al.^7^ applied Mahalanobis distance discriminant analysis (DDA) method to establish a DDA model for CBL classification based on 95 groups of training samples.

The above studies have achieved a series of good results in CBL classification, but are limited to mathematical modelling under small samples, the approach to problem-solving is complex and subjective, and the learning ability of model is poor. So, it is difficult to meet the convenience and intelligence requirements of current CBL classification. In addition, the existing performance evaluation indicator of classification model is single, the accuracy is used chiefly as the main indicator, which only considers overall classification effect, and it is difficult to ensure the CBL classification effect of different levels. In recent years, machine learning methodologies, such as BPNN^19,20^ and SVM^21,22^ have been increasingly employed to address various challenges within the domain of mining engineering. These advanced techniques have demonstrated notable efficacy in tackling analogous issues encountered in this field. Aiming at the shortcomings of CBL classification, this paper established an extensive sample dataset of CBL, introduced machine learning methods to carry out intelligent classification research of bursting liability, considered the CBL classification effect of different levels, and comprehensively used the accuracy, F1-score and Kappa coefficient to evaluate classification performance and select the best learning model.

Based on the above explanation, this study presents several novel contributions to the CBL classification:

- 1. The largest dataset comprises an extensive collection of samples sourced from pertinent literature sources. This dataset encompasses 127 distinct data sets, systematically incorporating stress and energy variables into its framework.
- 2. Nine unconstrained optimization algorithms were integrated with BP neural networks, and the SVM models were optimized utilizing the APSO algorithm. Subsequently, hybrid models were developed for comprehensive research in CBL.
- 3. By conducting a thorough comparison of accuracy, F1-score, and Kappa coefficient, alongside a sensitivity analysis of pertinent indicators, a refined optimization mechanism tailored to the CBL model was formulated.

## Methods

### Overview of CBL

CBL characterizes the ability of coal to store strain energy and experience impact-induced fractures, essential for predicting coal burst incidents. Classified into three levels—none, weak, and strong—CBL’s intensity directly influences the probability of coal bursts. The stronger the CBL, the higher the likelihood of coal burst occurrences. The CBL evaluation is typically determined through a comprehensive analysis of various laboratory-determined discriminative indexes. Commonly used indexes include the duration of dynamic fracture (*DT*), elastic strain energy index (*W*_ET_), bursting energy index (*K*_E_), and uniaxial compressive strength (*R*_C_).

**Figure 1:**
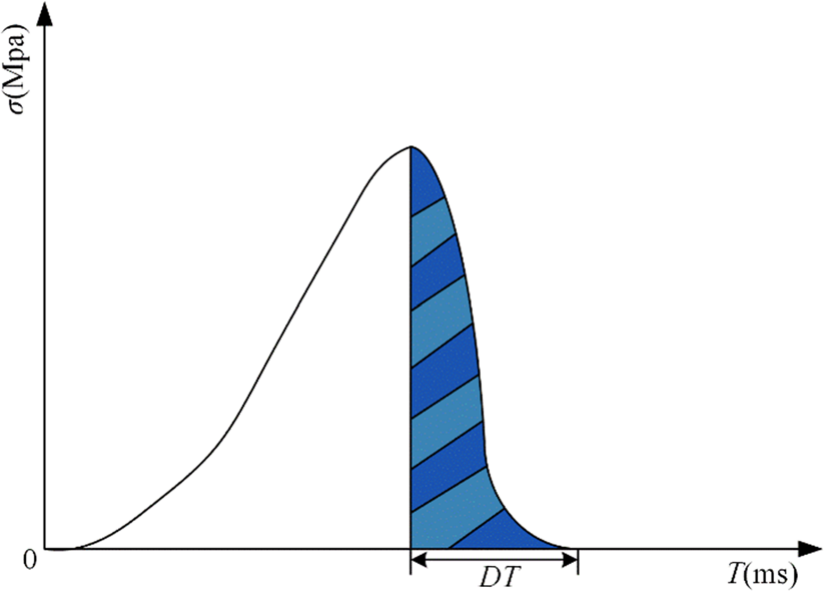
*DT* schematic diagram.

DT is defined as the period required for a coal sample under uniaxial compression to proceed from its ultimate strength to total failure, as illustrated in Fig. 1. A shorter failure time indicates a stronger CBL.

When calculating *DT*, the average value of the dynamic failure time for each group of samples should be taken, as shown in Eq. (1).

1 $$DT = \frac{1}{n}\sum\nolimits_{i = 1}^{m} {DT_{i} } ,$$

where *DT* is the average dynamic failure time (ms), *DT*_*i*_ is the dynamic failure time of the *i*-th specimen (ms), *n* is the number of specimens per group.

**Figure 2:**
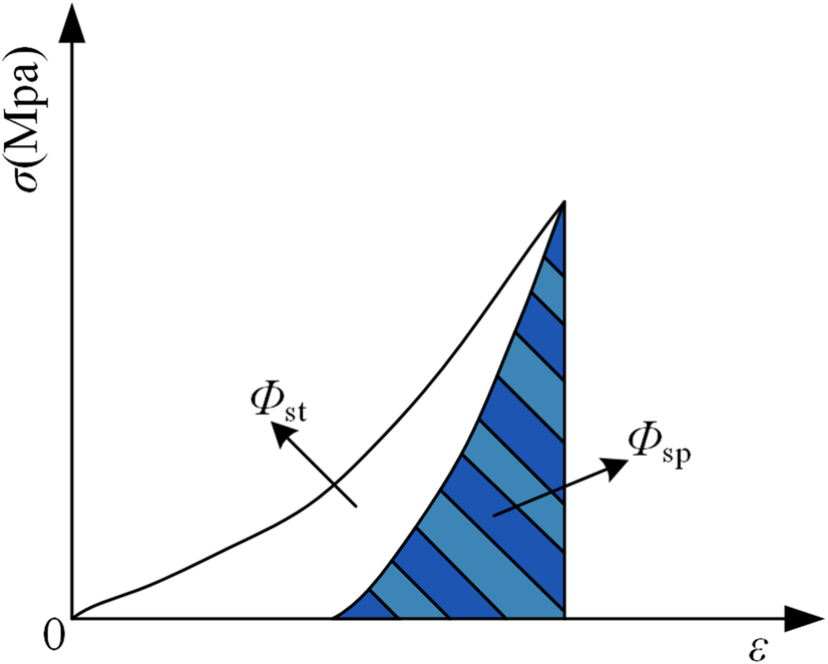
*W*_ET_ schematic diagram.

(2) *W*_ET_ is defined as the quotient of elastic deformation energy to plastic deformation energy (dissipated deformation energy) in a coal sample under uniaxial compression before it reaches a specific stress threshold (prior to failure), as depicted in Fig. 2. An increase in the *W*_ET_ value signifies a stronger CBL. The formula for calculating *W*_ET_ is presented in Eq. (2).

2 $$W_{{{\text{ET}}}} = \Phi_{{{\text{sp}}}} /\Phi_{{{\text{st}}}}$$

**Figure 3:**
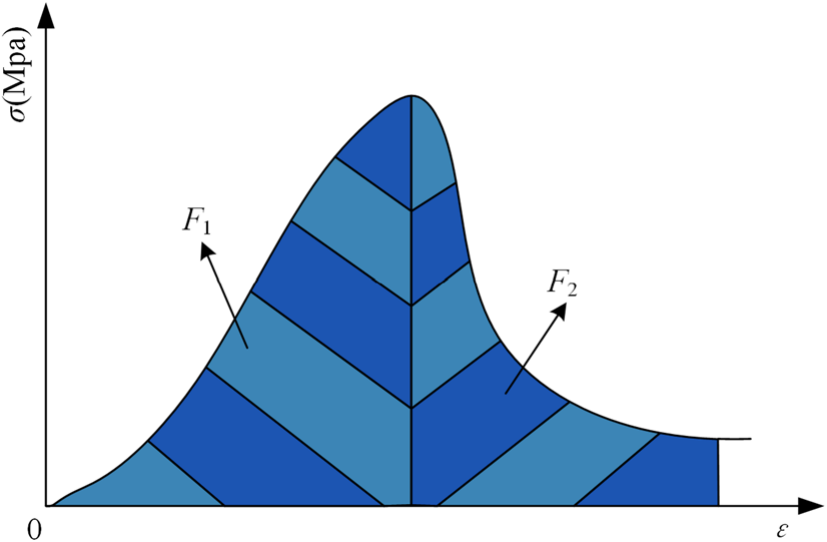
*K*_E_ schematic diagram.

(3) *K*_E_ is articulated as the proportion of accumulated deformation energy (*F*_1_) preceding peak stress to the dissipated deformation energy (*F*_2_) subsequent to peak stress, as evidenced in the stress-strain curve of a coal sample under uniaxial compression, illustrated in Fig. 3. An elevated *K*_E_ value delineates a stronger CBL, indicating a higher predisposition towards coal burst events. The calculation formula for *K*_E_ is given in Eq. (3).

3 $$K_{{\text{E}}} = F_{{1}} /F_{{2}}$$

(4) *R*_C_ is defined by the quotient of the failure load (*Q*) to the bearing surface area (*A*) of a standardized coal sample subjected to uniaxial compression. This metric, illustratively presented in Eq. (4), serves as a pivotal indicator of CBL, where an augmented *R*_C_ value signals an increased risk to coal burst.

4 $$R_{{\text{C}}} = Q/A$$

### Machine learning methods

In recent years, machine learning methods have been widely used in the classification and evaluation of engineering problems. The ‘black box’ nature of machine learning models allows them to circumvent complex mechanistic issues in decision-making, thereby attracting significant attention. Machine learning research began with the introduction of neural networks^23^, nearly 80 years, perceptron^24^, Hubel-Wiesel biological visual model^25^, back propagation neural network (BPNN)^26^, decision tree^27^, support vector machine (SVM)^28^, boosting^29^ and other models have been developed, which have become the mainstream of machine learning applications.

Index selection is essential in studying of CBL classification by machine learning methods. Referring to previous studies^7,16,30^, duration of dynamic fracture (*DT*), elastic strain energy index (*W*_ET_), bursting energy index (*K*_E_), and uniaxial compressive strength (*R*_C_) as the classification indexes were selected and divided the CBL level into three classes: strong (class 1), weak (class 2), and none (class 3), and they are considered as the output of machine learning model.

As the most representative machine learning methods, BPNN and SVM are adopted for CBL classification in this paper. The best model is selected by establishing different machine learning models and evaluating their performance. A total of twelve machine learning models are established, including ten BPNN models and two SVM models.

Ten BPNN models include one standard model based on gradient descent and nine improved models according to different unconstrained optimization algorithms (quantized conjugate gradient method, Fletcher-Reeves conjugate gradient method, Polak-Ribiere conjugate gradient method, momentum gradient descent method, adaptive lr gradient descent method, adaptive lr momentum gradient descent method, elastic gradient descent method, Levenberg‒Marquardt algorithm, and quasi-Newton algorithm). Principles of these algorithms are described in references^30–32^. Table 1 shows the ten BPNN models based on different algorithms.

**Table 1:** Different BPNN models.

No.	Trained method	BPNN model
1	Gradient descent	Standard BPNN
2	Quantized conjugate gradient	SCG-BPNN
3	Fletcher-Reeves conjugate gradient	CGF-BPNN
4	Polak-Ribiere conjugate gradient	CGP-BPNN
5	Momentum gradient descent	GDM-BPNN
6	Adaptive lr gradient descent	GDA-BPNN
7	Adaptive lr momentum gradient descent	GDX-BPNN
8	Elastic gradient descent	RP-BPNN
9	Levenberg–Marquardt	LM-BPNN
10	Quasi-Newton	BFG-BPNN

The two SVM models established in this paper, one is linear SVM (LSVM) model, which transforms the constrained problem of soft margin maximization into an unconstrained problem using the Lagrange function. The other is the APSO-SVM model, which uses an adaptive particle swarm optimization algorithm (APSO) to optimize the SVM model. This algorithm improves the traditional particle swarm optimization algorithm, and the specific principle can be found in the reference^33^.

### Data standardization

Data standardization is dimensionless, which can eliminate the influence of different dimensions and value ranges of indexes on classification results. In this paper, unified extreme value processing method is selected for data standardization. The principle is shown in Eq. (5).

5 $$\begin{gathered} x_{ij}^{*} = \frac{{x_{ij} - \min \left( {x_{j} } \right)}}{{\max \left( {x_{j} } \right) - \min \left( {x_{j} } \right)}} \hfill \\ \left( {i = 1,2,...,m;j = 1,2, \cdots n} \right) \hfill \\ \end{gathered}$$

where *x*_*ij*_ is the original data of *j*-th index of *i*-th sample; *x*_ij_^*^ is the processed data, whose value is between 0 and 1, and the data distribution is consistent with that of before processed; max(*x*_*j*_) and min(*x*_*j*_) are the maximum and minimum value of original data of index *j*, respectively.

### Performance evaluation

In this paper, three indicators, the accuracy, F1-score, and Kappa coefficient, are used to evaluate classification performance of different machine learning models comprehensively and scientifically.

(1) The accuracy^34^ is the ratio of the number of simples for accurate classification to the number of samples for modeling. The calculation formula for accuracy is given in Eq. (6).

6 $$Accuracy = \frac{TP + TN}{{TP + TN + FP + FN}},$$

where *TP* is the true positive in confusion matrix; *FP* is the true positive in confusion matrix; *FN* is the false negative in confusion matrix; *TN* is the true negative in confusion matrix.

**Figure 4:**
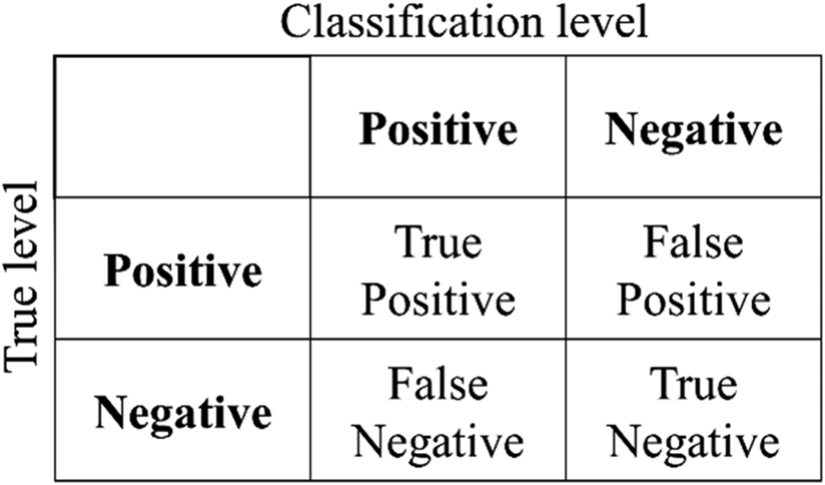
Confusion matrix schematic.

The confusion matrix is shown in Fig. 4.

(2) F1-score^35^ is an effective indicator to measure the classification accuracy. It considers the accuracy and recall of the classification model and is a harmonic average method of the accuracy and recall of the model. The value of F1-score is between 0 and 1, and the closer to 1, the stronger the comprehensive classification ability of the classifier. The calculation formula for F1-score is given in Eqs. (7–9).

7 $$F1 - score = \frac{2Precision \times Recall}{{Precision + Recall}},$$

8 $$Precision = \frac{TP}{{TP + FP}},$$

9 $$Recall = \frac{TP}{{TP + FN}},$$

where the *Precision* is the proportion of positive examples in the model, and *Recall* is the proportion of predicted positive examples in the total positive examples.

(3) Kappa coefficient^36^ is a parameter calculated based on confusion matrix in statistics to measure the classification accuracy. The value of Kappa coefficient is close to 1, indicating that the actual output and classified output have strong consistency. The calculation formula for Kappa coefficient is given in Eqs. (10) and (11).

10 $$Kappa = \frac{{Accurary - p_{e} }}{{1 - p_{e} }},$$

11 $$p_{e} = \frac{{\left( {TP + FP} \right) \cdot (TP + FN) + (FP + TP) \cdot (FP + TN)}}{{\left( {TP + FP + FN + TN} \right)^{2} }},$$

where the *p*_*e*_ is the total probability the raters both saying positive and negative randomly.

### Research route

**Figure 5:**
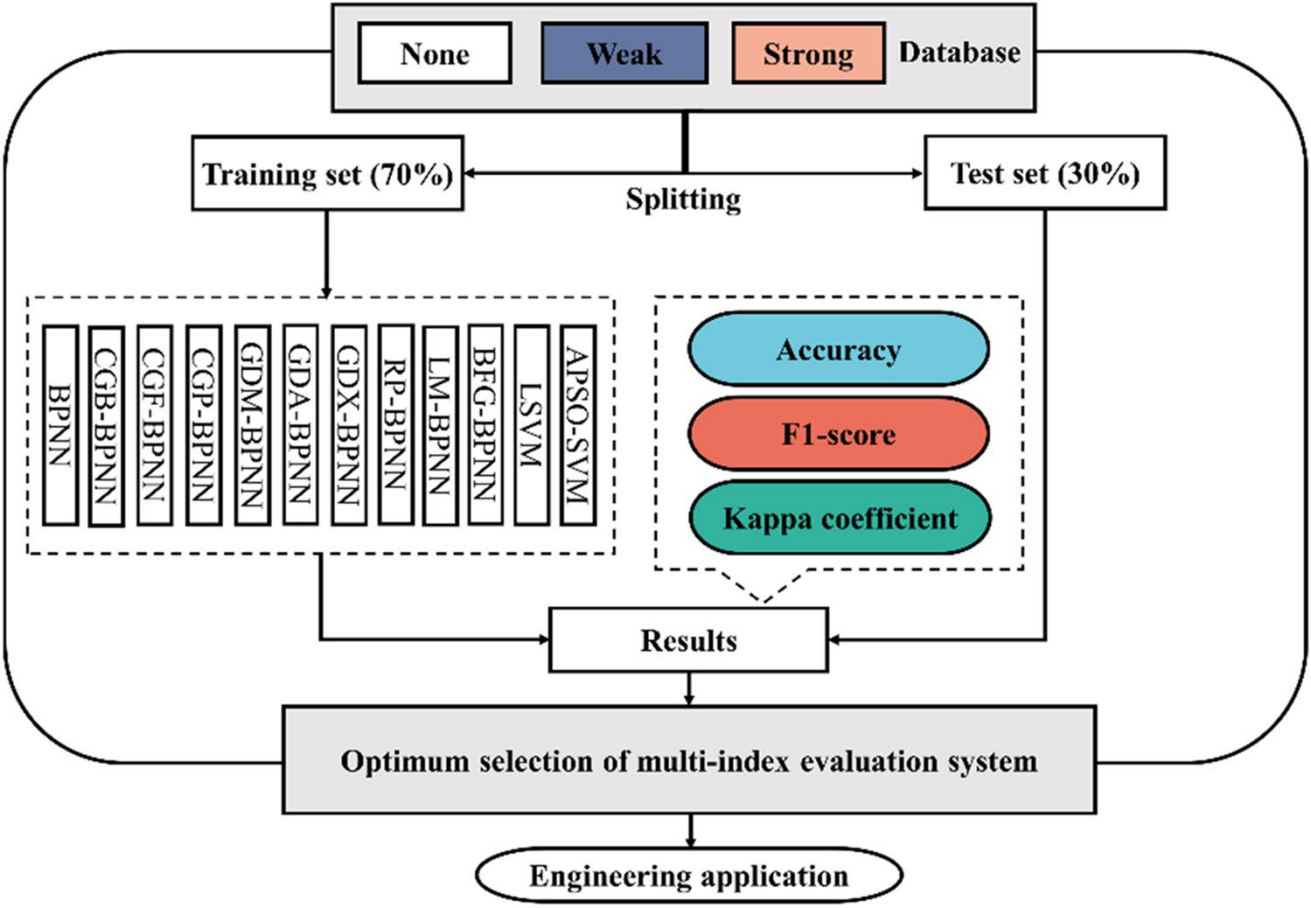
Research flowchart.

In this paper, firstly, an imbalanced sample set consisting of 127 groups of CBL samples is established, and the sample set is divided into a training set and a test set according to the ratio of 7:3. Then, twelve machine learning models are adopted to classify the samples, and the classification results are evaluated and analyzed for model optimization, and ultimately, the best model is applied to engineering contexts. The research flowchart is shown in Fig. 5.

### CBL classification using ML methods

#### Data analysis

**Figure 6:**
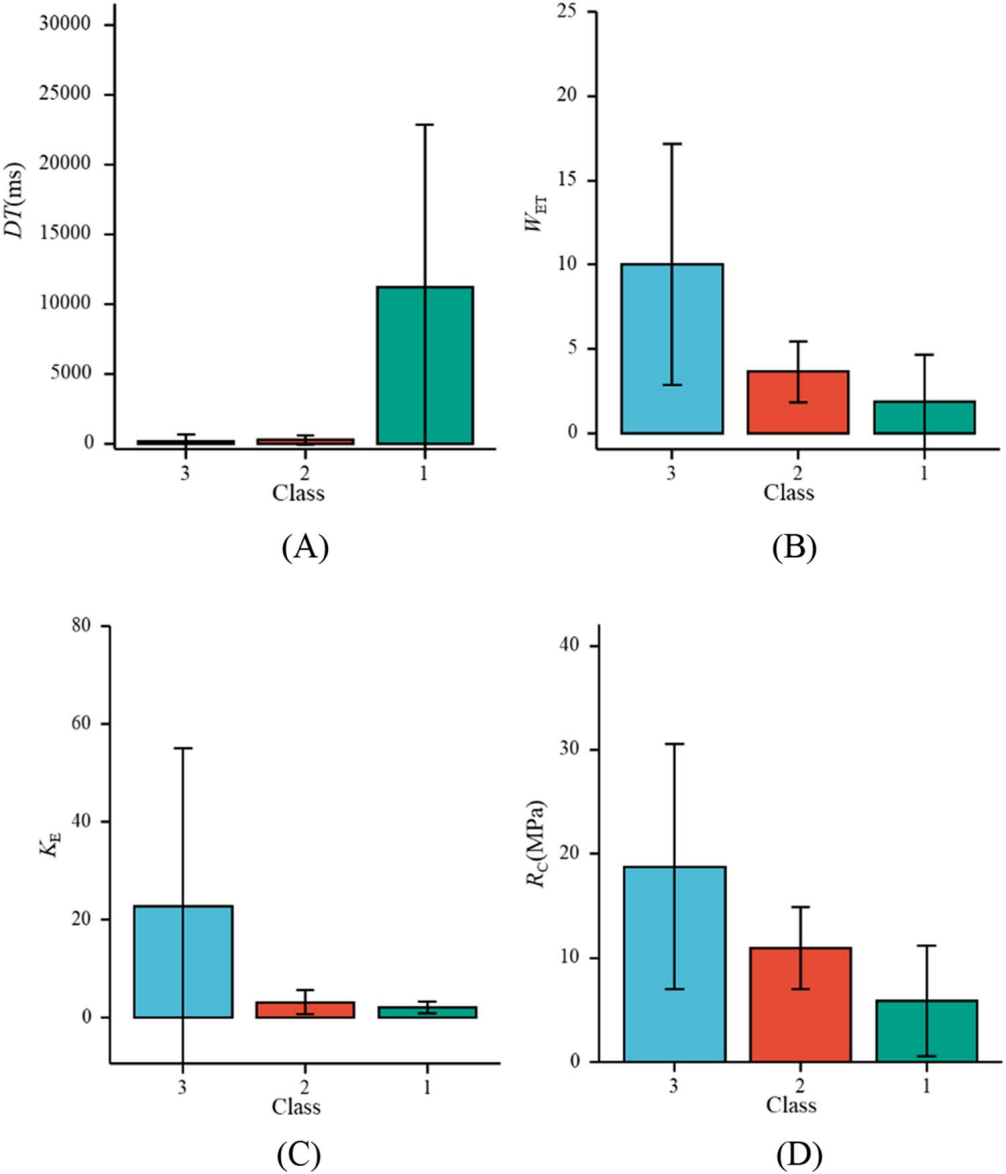
Box diagram of CBL indexes. (**A**) *DT*. (**B**) *W*_ET_. (**C**) *K*_E_. (**D**) *R*_C_.

By consulting a large number of literatures, 127 well-documented CBL samples have been collected (All data represent the average values obtained from multiple coal samples in this group, with specific details provided in the Supplementary Materials.), including 53 strong bursting liability samples, 67 weak bursting liability samples, and 7 none bursting liability samples. Figure 6 shows the data distribution of *DT*, *W*_ET_, *K*_E_, and *R*_C_ with different bursting liabilities. Table 2 presents in detail the statistical characteristics of the indexes data. It can be seen that the data of each index is evenly distributed without obvious abnormal values, and the index selection is reasonable.

**Table 2:** Statistical characteristics of index data.

Index	Maximum	Minimum	Median	Standard deviation	Median
*DT* (ms)	31,325	3.75	843.608	3597.692	156
*W*_ET_	49	0.2	6.208	5.843	4.45
*K*_E_	123	0.87	10.946	21.943	4.13
*R*_C_ (MPa)	72.45	1.56	14.138	9.139	12.52

**Figure 7:**
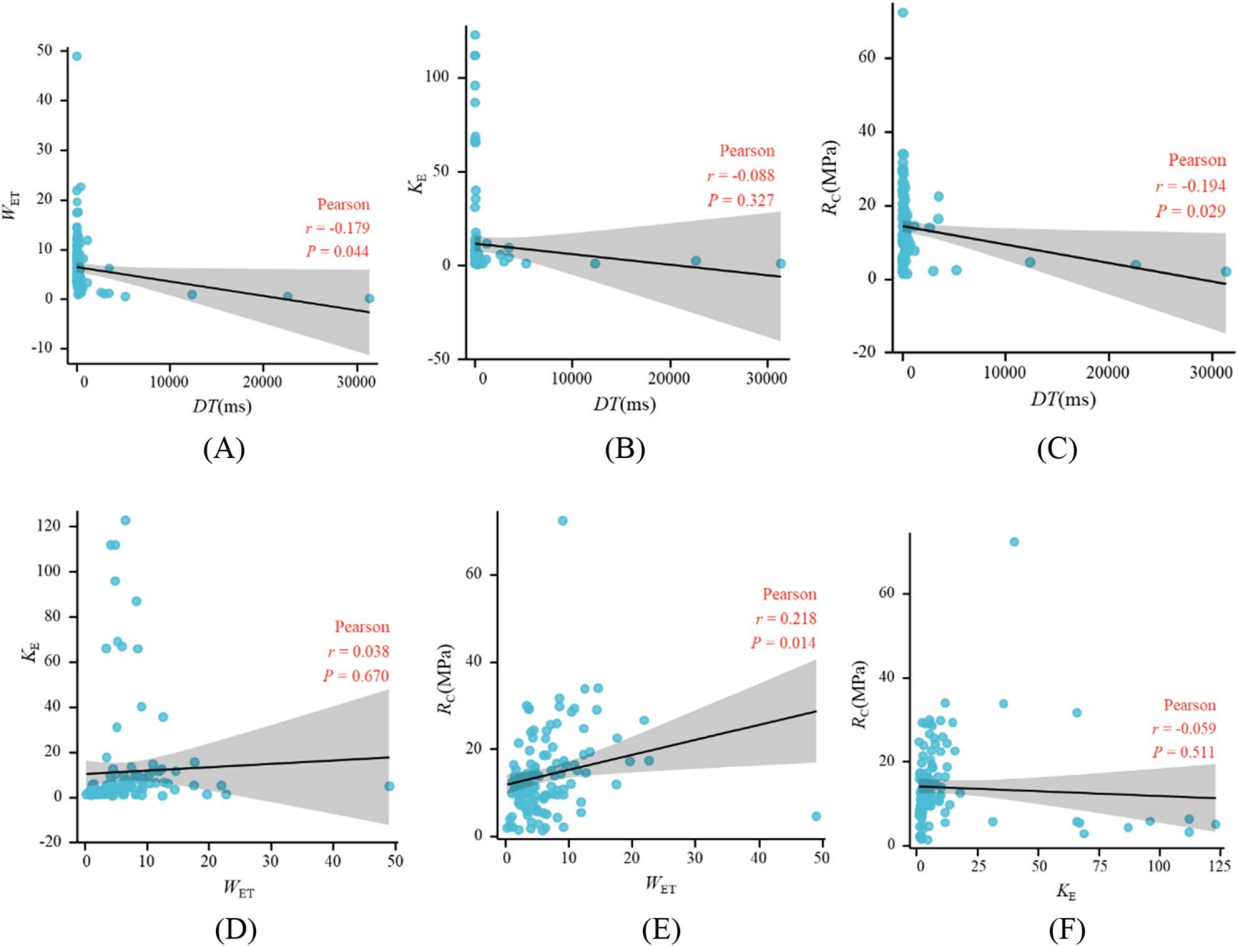
Correlation analysis of indexes. (**A**) *DT* and *W*_ET_. (**B**) *DT* and *K*_E_. (**C**) *DT* and *R*_C_. (**D**) *W*_ET_ and *K*_E_. (**E**) *W*_ET_ and *R*_C_. (**F**) *K*_E_ and *R*_C_.

In order to ensure the rationality of modelling, further correlation analysis is required. The Pearson correlation coefficient, a widely used measure of correlation in various fields, serves as an index of correlation analysis. Its value is between −1 and 1, which can measure the linear correlation between the two indexes. The greater the absolute value of correlation coefficient, the stronger the correlation between indexes. Figure 7 shows the correlation analysis of *DT*, *W*_ET_, *K*_E_, and *R*_C_, including significant level (*P*), correlation coefficient (*r*) and curve fitted by the correlation coefficient. When analyzing the correlation of indexes, it is necessary to test their significance level first. After passing the significance test, the correlation coefficient can be used for correlation analysis. Figure 7 shows the CBL classification indexes have weak or no correlation, indicating the selected indexes are scientific and reasonable.

**Figure 8:**
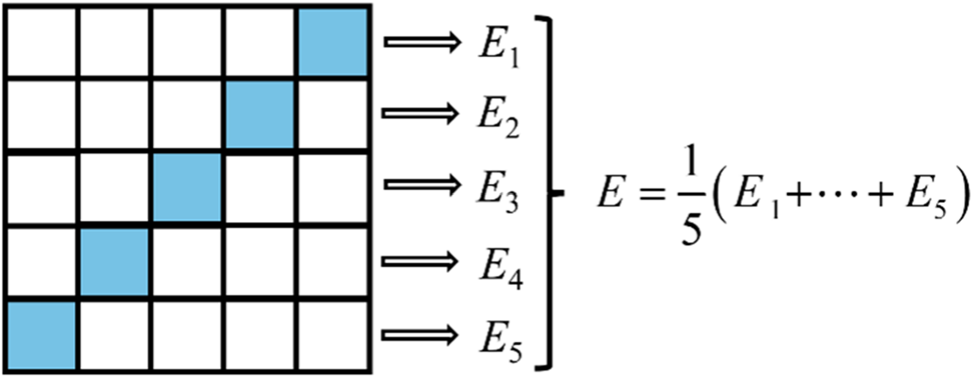
fivefold cross-validation.

Based on it, the database, containing 127 groups of samples, was divided into a training set (70%) and a test set (30%), and the data were tested by using fivefold cross-validation, as shown in Fig. 8. Professional data analysis software is adopted to process data and construct models in this paper.

#### Model construction

##### BPNN

**Figure 9:**
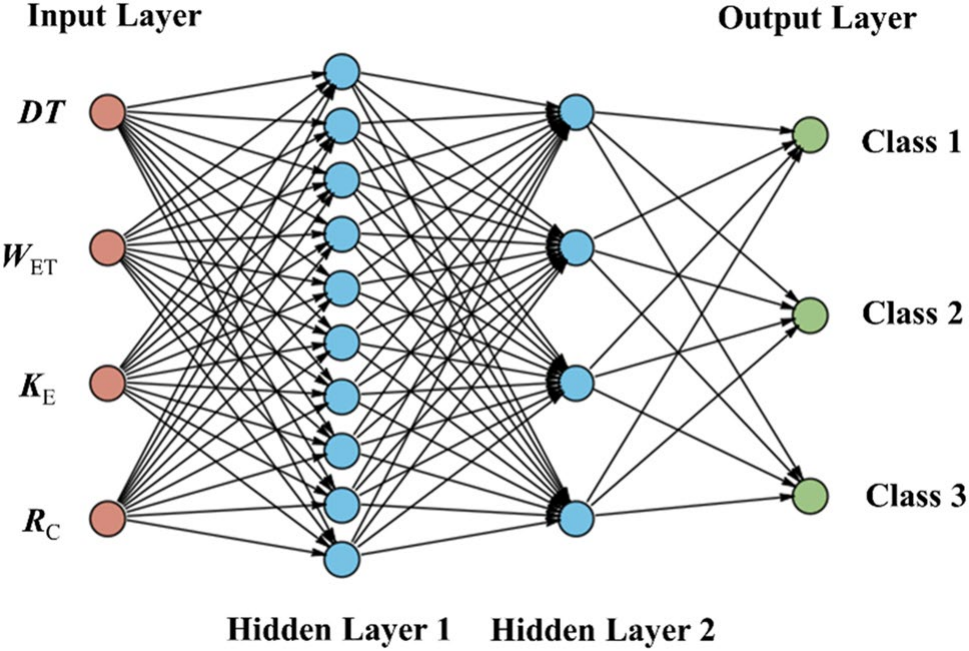
Structure of BPNN.

BPNN is a model proposed by Rumelhart and McClelland^37^. The input layer of BPNN built in this paper has four neurons, namely, *DT*, *W*_ET_, *K*_E_, and *R*_C_. The configuration of the hidden layer is based on the guidelines provided in references^38,39^. Two hidden layers are set, and the number of hidden layer nodes selected after multiple debugging is 10 and 4, respectively. The output layer of BPNN has three neurons, corresponding to the class 1 to 3 of CBL level, as shown in Fig. 9.

**Figure 10:**
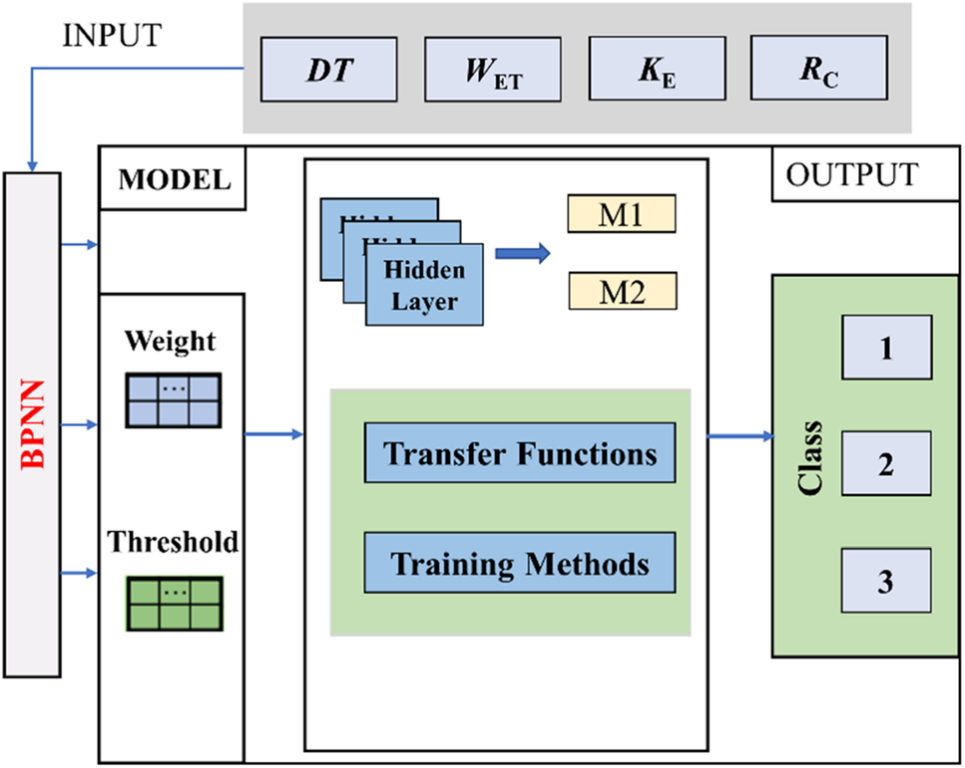
BPNN for CBL classification.

The upper limit of the training times of the ten BPNN models built in this paper is set to 1000, the learning rate is 0.01, the minimum error of the training target is 0.0001, and the display frequency is 25, that is, it is displayed once every 25 times of training, the minimum performance gradient is 1*e−6, and the maximum number of failures is 6. The operation process of the model is shown in Fig. 10.

##### SVM

**Figure 11:**
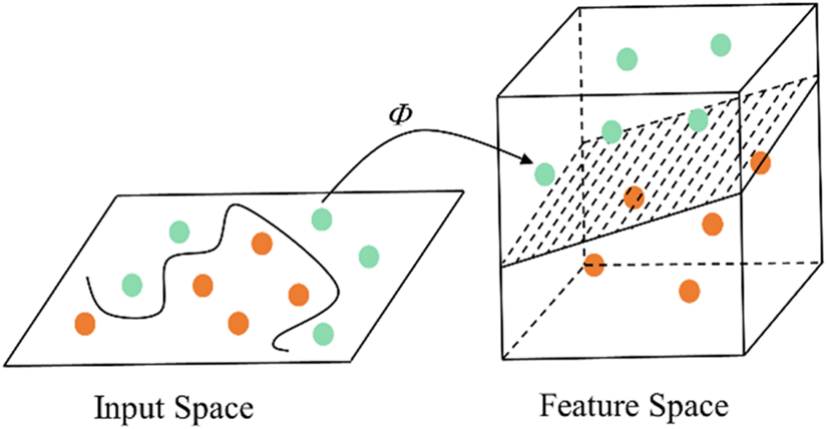
Classification principle of SVM.

SVM^40,41^ is a supervised learning algorithm proposed by Vapnik and other scholars to analyze data and classify decisions. Based on the statistical learning theory and structural risk minimization principle, SVM uses the limited sample information to establish a classification hyperplane in the feature space for obtaining classification and recognition abilities. It offers advantages such as requiring fewer samples, handling nonlinearity, and functioning effectively in high-dimensional spaces. In the classification process, SVM maps the low-dimensional data to the high-dimensional space through the higher-dimensional space of kernel function^26^, as shown in Fig. 11.

As for the selection of model parameters, the penalty factor *c* of LSVM model is 1.0, the kernel function is linear kernel function, and the parameter *g* is set to the reciprocal of the number of sample features. APSO-SVM model selects the RBF radial basis kernel function, penalty factor *c* and kernel function parameter *g* adopt APSO for parameter optimization, and other parameters are the same as LSVM model.

## Results

### Classification result and evaluation

**Figure 12:**
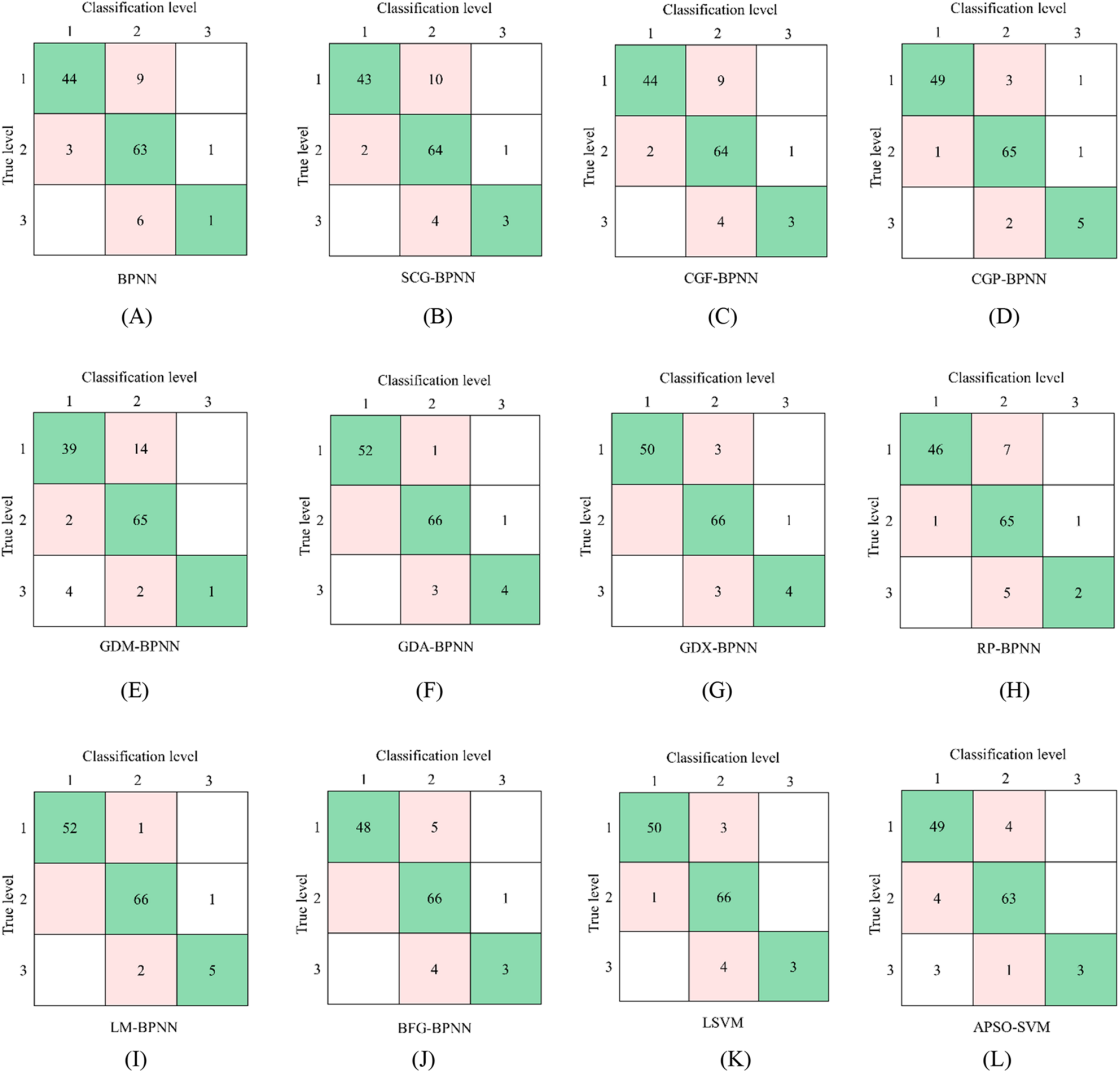
Confusion matrix of twelve models. (**A**) BPNN. (**B**) SCG-BPNN. (**C**) CGF-BPNN. (**D**) CGP-BPNN. (**E**) GDM-BPNN. (**F**) GDA-BPNN. (**G**) GDX-BPNN. (**H**) RP-BPNN. (**I**) LM-BPNN. (**J**) BFG-BPNN. (**K**) LSVM. (**L**) APSO-SVM.

For the multi-index and multi-classification problem, the accuracy, F1-score, and Kappa coefficient were used as the evaluation indicators in this paper. The accuracy can better reflect the overall classification performance of model, and the other two indicators can better reflect the classification accuracy of model. Based on this, the confusion matrixes are constructed for twelve learning models (see Fig. 12), and the values of three evaluation indicators of each model are calculated, as shown in Table 3.

**Table 3:** Values of performance evaluation indicators of twelve models.

No.	Model	Accuracy (%)	F1-score	Kappa coefficient
1^#^	BPNN	85.83	0.6885	0.7279
2^#^	SCG-BPNN	86.61	0.7830	0.7456
3^#^	CGF-BPNN	87.40	0.7882	0.7610
4^#^	CGP-BPNN	93.70	0.8720	0.8454
5^#^	GDM-BPNN	81.89	0.7220	0.6485
6^#^	GDA-BPNN	96.06	0.8787	0.9256
7^#^	GDX-BPNN	94.19	0.8680	0.8913
8^#^	RP-BPNN	88.98	0.7641	0.7899
9^#^	LM-BPNN	96.85	0.9113	0.9417
10^#^	BFG-BPNN	85.36	0.8217	0.7122
11^#^	LSVM	93.70	0.8648	0.8816
12^#^	APSO-SVM	90.55	0.7639	0.7279

### Analysis and optimization

#### Analysis

1. Classification performance analysis

According to Table 3, the LM-BPNN model has the highest accuracy rate (96.85%), the accuracy rate (81.89%) of GDM-BPNN model is lowest, and the average accuracy rate of twelve models is 90.09%. It can be seen that the accuracy rate of CBL classification model constructed in this paper is high, but there is a large gap between the accuracy of models based on the same machine learning method and different optimization algorithms.

Unlike the accuracy, F1-score focuses more on identifying the data with the actual sample level, which is a measurement standard that combines the accuracy rate and recall rate. Table 3 shows that the LM-BPNN model has a highest F1-score value (0.9113), the standard BPNN model has a lowest F1-score value (0.6885), and the average F1-score value of twelve models is 0.8109. Compared with the accuracy, F1 score considers the situation of samples with different levels. For example, the accuracy of RP-BPNN model is 88.98%, however, the classification effect of samples of class 3 is poor (only two groups of samples are correct) seen from the confusion matrix, and the F1 score value is only 0.7641, which is lower than that of the CGF-BPNN model with an accuracy rate of 87.40%.

Kappa coefficient is also an evaluation indicator under imbalanced samples. Different from F1-score, Kappa coefficient focuses more on categories with a small sample size. Table 3 shows that the Kappa coefficient value (0.9417) of LM-BPNN is highest, the Kappa coefficient value (0.6485) of GDM-BPNN is smallest, and the average value of twelve models is 0.7999. Compared with the accuracy rate and F1-score value, the fluctuation of Kappa coefficient of different models is more obvious.

**Figure 13:**
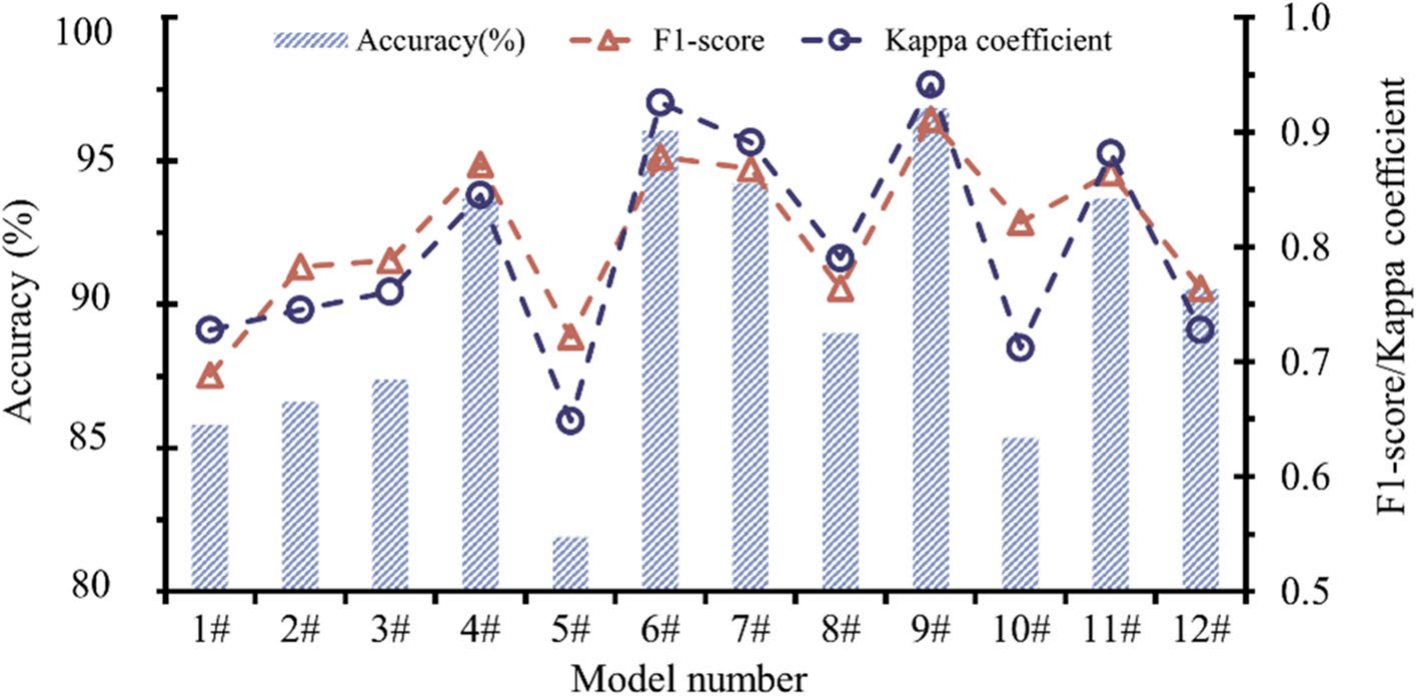
Classification performance of twelve models.

Figure 13 shows the change curve of the accuracy rate, F1-score value, and Kappa coefficient value of twelve classification models. It can be seen that the overall trend of three change curve is similar, among which the accuracy curve is the most stable. The Kappa coefficient is similar to the accuracy trend, but the fluctuation is largest, which is determined by design intention and calculation method of Kappa coefficient. The accuracy is directly involved in the calculation of Kappa coefficient, so it is closer in the overall trend. The design of Kappa coefficient is to consider the categories with few samples. Among the three types of CBL samples, there are fewer class 3 samples, so the classification results of class 3 samples have a greater impact on the Kappa coefficient value and are prone to obvious fluctuations. F1-score not only considers the imbalanced sample set, but also does not pay special attention to a certain category of samples, which is more balanced than the other two evaluation indicators.

(2) Analysis of model characteristics

BPNN model is a multilayer feedforward neural network model. The gradient descent method and nine unconstrained optimization algorithms were selected to train BPNN models. Different algorithms result in different classification effects. As an optimization algorithm of differentiable function, gradient descent method solves the learning problem of multi-layer neural network, but it also has some shortcomings. It can be seen from Table 3 that the classification accuracy of standard BPNN model is lower than that of most improved BPNN models, and also lower than the average accuracy of twelve models.

The nine improved BPNN models were established according to different unconstrained optimization algorithms. As far as the classification effect is concerned, they present positive and negative optimization, that is, whether the classification effect is better than the standard BPNN model. GDM-BPNN model and BFG-BPNN model show negative optimization, in which GDM-BPNN model adds a momentum factor in the weight updating stage of gradient descent method to strengthen the anti-vibration ability of the model. The classification results indicate that improving the model’s anti-vibration ability does not correlate with, or enhance, CBL classification accuracy. BFG-BPNN model is established based on quasi-Newton method. Compared with gradient descent method, this method optimizes the convergence speed of BPNN, but the gradient of objective function needs to be known when calculating, the model established in this paper is not given, resulting in low accuracy.

The other seven improved BPNN models show positive optimization. Due to optimization angles of different unconstrained optimization algorithms are different, three improved BPNN models with highest classification accuracy were analyzed in this paper, namely, LM-BPNN model, GDA-BPNN model, and GDX-BPNN model. LM-BPNN model is a BPNN model optimized according to the Levenberg–Marquardt algorithm. Levenberg–Marquardt algorithm is a classical trust region algorithm, which optimizes the Newton method most commonly used in optimization problems, and also optimizes the complex computational problems in quasi-Newton method, so it has the advantages of gradient method and Newton method at the same time. The classification results show that the accuracy of LM-BPNN model is 96.85%, which is the highest among all models. GDA-BPNN model and GDX-BPNN model are optimized based on adaptive lr gradient descent method and adaptive lr momentum gradient descent method, respectively. The optimization of traditional gradient descent method is mainly reflected in the adaptive learning rate. This method adapts to each parameter in the update process and determines the large or small update amount according to the importance of each parameter, so that the algorithm has a faster convergence speed and better classification accuracy. The classification results of this paper also verify the advantages of adaptive learning rate method.

For the two SVM models established in this paper, from the classification results, the performance of LSVM model using linear kernel function is better than APSO-SVM model. The correlation between classification indicators has been verified above, so LSVM model is more suitable for the sample database built in this paper. APSO algorithm is an improved particle swarm optimization algorithm. The APSO-SVM model established in reference^33^ is a classification model applicable to nonlinear data. Through the analysis of principle and results, the sample database built in this paper is more suitable for linear models or models that do not require index correlation.

### Model optimization

In order to select the best machine learning model from the twelve models, the following optimization rules were developed in this paper: Since larger values in the three performance evaluation indicators signify better performance, priority is given to the classification model with the highest values. When the values of evaluation indices are similar, preference is given to models with superior stability and suitability for the established sample database.

Through comprehensive comparative analysis, it can be seen that the accuracy (96.85%) of LM-BPNN model is the highest, representing the best overall classification accuracy; F1 score value (0.9113) and Kappa coefficient value (0.9417) are also the highest, representing that the CBL classification results of different classes are good, which can be verified by the confusion matrix of the model. In summary, LM-BPNN model is selected as the best model in this paper.

### Sensitivity analysis

**Figure 14:**
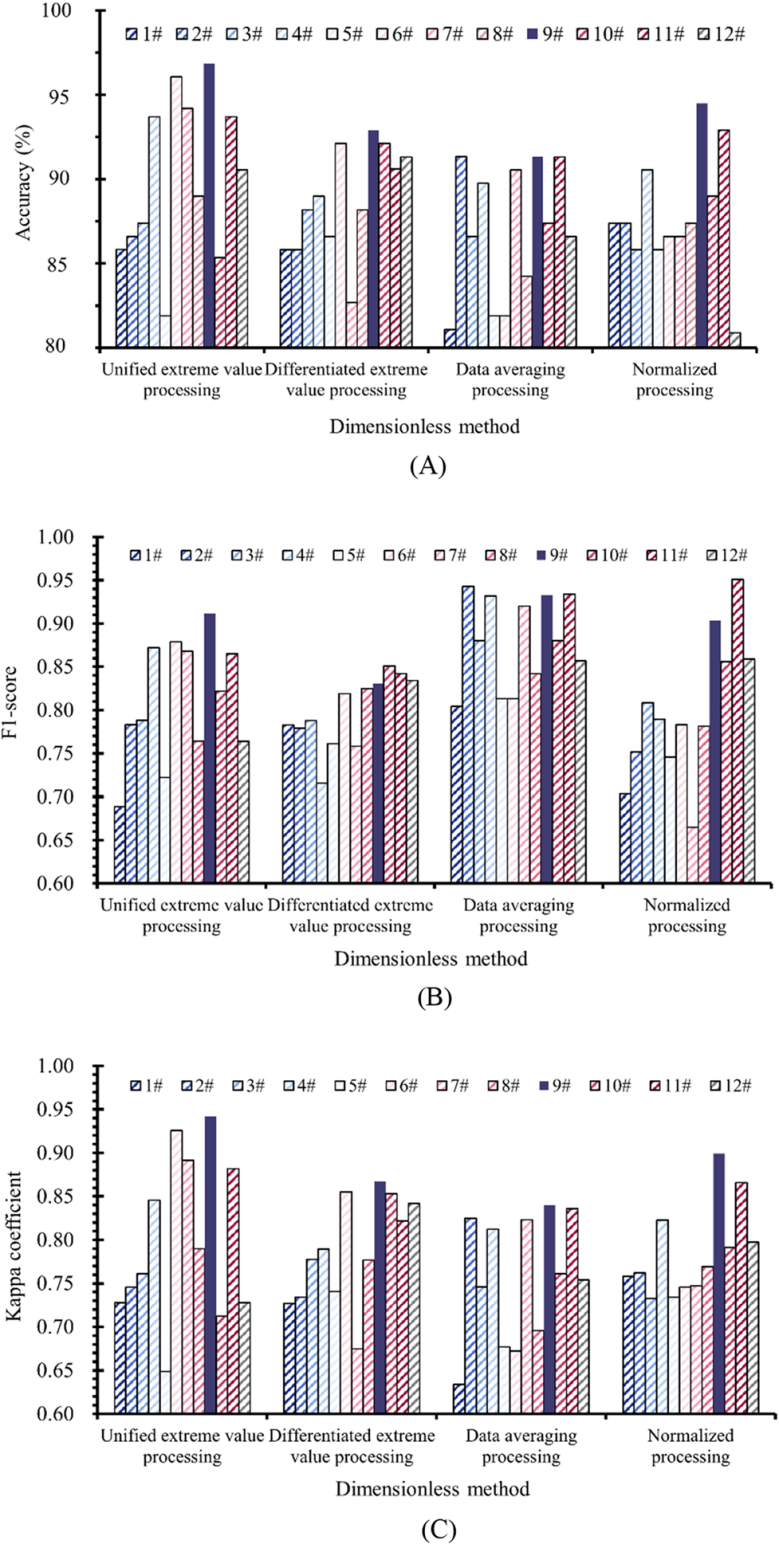
Model performance evaluation. (**A**) Accuracy. (**B**) F1-score. (**C**) Kappa coefficient.

In the sphere of machine learning, the initial phase of model deployment is underscored by data preprocessing, a critical procedure adopted across a spectrum of machine learning methodologies within mining engineering^41–45^ to bolster model precision and reliability. This investigation pursues the identification of the superior model via a sensitivity analysis centered on data preprocessing techniques specific to index data. Through the application of diverse preprocessing strategies, including differentiated extreme value processing, averaging processing, and normalized processing, sample data is transformed into a dimensionless format. The classification results of the models built using these methods are compared with the results obtained using the unified extreme value processing method in data analysis. The performance evaluation results of the models can be found in Fig. 14.

Referring to Fig. 14, the LM-BPNN model (No.9#), selected for this investigation, demonstrates commendable performance across all four dimensionless data preprocessing techniques. The model's three evaluative metrics—Accuracy, F1-score, and Kappa coefficient—register at comparably elevated tiers, underscoring the model’s robustness to variations in data preprocessing and its enhanced classification consistency. Further analysis, as depicted in Fig. 14c, reveals that the LM-BPNN model consistently secures the apex position in terms of the Kappa coefficient value across disparate data processing strategies. This evidences the model’s aptitude for handling imbalanced sample databases effectively. The study’s sample database, characterized by a significant imbalance with a distribution ratio of 53:67:7 across three sample categories, benefits from the model’s superior ability to classify samples belonging to the less represented category, specifically the coal samples categorized under weak CBL, thereby outshining other models under comparison.

### Engineering application

To verify the engineering application performance, LM-BPNN model is utilized to Wudong Coal Mine and N2105 working surface of a coal mine in Yvwu Coal Industry.

1. Wudong coal mine

Wudong Coal Mine^46^ is located in the north and south wings of Badaowan syncline in Urumqi mining area, China. The mining level of the coal seam is +475 and the mining depth is about 375 m. The immediate roof of the B3 + 6 coal seam consists of grey argillaceous rocks, with a thickness ranging from 1.5 to 2.1 m. It has a blocky structure and becomes loose when exposed to water, leading to collapse in the later stages of mining. The basic roof is composed of siltstone, with a thickness of 24.49 m. It is characterized by its hard and stable nature and does not collapse during mining operations. The immediate floor is composed of charcoal mudstone, with an average thickness of 1.35 m. It has the potential to collapse in the later stages of mining. The basic floor consists of fine-siltstone. LM-BPNN model is applied to the CBL classification of five groups of coal in Wudong Coal Mine, and the classification results are consistent with the actual impact dynamics of the coal seam (the coal seam has experienced three times of coal burst during mining), as shown in Table 4 for details.

(2) N2105 working surface of a coal mine in Yvwu coal industry

**Table 4:** CBL classification of Wudong coal mine.

No.	*DT* (ms)	*W*_ET_	*K*_E_	*R*_C_ (MPa)	Engineering guidance level	LM-BPNN	Sampling spot
1	119	4.55	2.95	15.25	2	2	B3 + 6 coal seam of +501 level
2	58	3.77	2.13	19.75	2	2	B3, B5 coal seam of +545 level
3	254	1.59	1.46	12.64	2	2	B6 coal seam of +545 level
4	391	4.64	0.9	5.81	2	2	B3, B5 coal seam of +400 level
5	33	3.63	2.25	29.35	1	1	B6 coal seam of +400 level

Yvwu Coal Mine is located in the middle east of Qinshui Coalfield, Shanxi Province, China. It is 16 km long from north to south and 10 km wide from east to west, with a total area of 161.205 km^2^. The burial depth of N2105 working face is 507–597 m. The average thickness of the coal seam being mined is 6.31 m, with a uniaxial compressive strength of 6.88 MPa. The main overlying roof consists of fine-siltstone sandstone, approximately 11 m thick. It has been identified that both the coal seam and its roof and floor have a weak CBL. After mining, the coal body is plastic damaged, and the bolt support is partially ineffective^47^. The classification results of LM-BPNN model are consistent with the actual situation on site. See Table 5 for details.

**Table 5:** CBL classification of N2105 working surface.

No.	*DT* (ms)	*W*_ET_	*K*_E_	*R*_C_ (MPa)	Engineering guidance level	LM-BPNN	Sampling spot
1	468	3.10	1.09	6.47	2	2	3# coal seam
2	313	2.65	1.35	6.88	2	2	

## Discussion

In this study, the largest database of CBL samples was created for classification research. The LM-BPNN model was selected from 12 CBL classification models. It is applied in Wudong Coal Mine and Yvwu Coal Mine. Setting itself apart from existing studies^15,17,18,48^, this paper not only expands the database but also diverges in research methodologies, manifested in three key points: Firstly, new technical means are introduced for CBL classification. Secondly, the ML model is optimized using unconstrained optimization algorithms and heuristic algorithms. Thirdly, a new evaluation mechanism suited for CBL classification is established through the integration of sensitivity analysis.

The research findings of this paper can be applied to mines requiring coal bursts control measures. It is important to note that the applicability of our model might vary, because there may be significant differences in coal mine conditions in production.

## Conclusions

In view of the shortcomings of traditional CBL classification methods, this paper selected *DT*, *W*_ET_, *K*_E_ and *R*_C_ as classification indexes, constructed a database containing 127 samples, and established twelve machine learning models based on BPNN and SVM for performance evaluation and optimization, and obtained the following conclusions:

- (1) Machine learning methods were introduced for CBL intelligent classification research for the first time, and the classification accuracy of the machine learning model established was between 81.89% and 96.85%.
- (2) The LM-BPNN model was identified as the most effective, demonstrating optimal performance among twelve models with a classification accuracy of 96.85%, an F1 score of 0.9113, and a Kappa coefficient of 0.9417. These metrics were used to assess the model’s classification capabilities across samples, supported by comprehensive analysis and sensitivity analysis.
- (3) The LM-BPNN model was applied to Wudong Coal Mine and N2105 working surface of a coal mine in Yvwu Coal Industry. The predictive results are consistent with actual conditions, yielding a new method for CBL classification with promising application prospects.

### Supplementary Information

Supplementary Information.

## Data Availability

All data generated or analysed during this study are included in this published article; further inquiries can be directed to the corresponding author.

## Abbreviations

CBL: Coal bursting liability
BPNN: Back propagation neural network
DDA: Distance discriminant analysis
*DT*: Duration of dynamic fracture
*W*_ET_: Elastic strain energy index
*K*_E_: Bursting energy index
*R*_C_: Uniaxial compressive strength
*N*: Number of specimens per group
*DT*_*i*_: Dynamic failure time of the* i*-th specimen
*Φ*_*sp*_: Elastic deformation energy
*Φ*_*st*_: Plastic deformation energy
*F*_1_: Accumulated deformation energy
*F*_2_: Dissipated deformation energy
*Q*: Failure load
*A*: Bearing surface area
SVM: Support vector machine
SCG: Quantized conjugate gradient
CGF: Fletcher-Reeves conjugate gradient
CGP: Polak-Ribiere conjugate gradient
GDM: Momentum gradient descent
GDA: Adaptive lr gradient descent
GDX: Adaptive lr momentum gradient descent
RP: Elastic gradient descent
LM: Levenberg–Marquardt
BFG: Quasi–Newton
LSVM: Linear support vector machine
APSO: Improved particle swarm optimization algorithm
*TP*: True positive in confusion matrix
*TN*: True negative in confusion matrix
*FP*: False positive in confusion matrix
*FN*: False negative in confusion matrix
*p*_*e*_: Parameters of kappa coefficient
*P*: Significant level
*r*: Pearson correlation coefficient
*c*: Penalty factor
*g*: Kernel function
GB/T: Recommended national standards in China

## Terminology interpretation

Coal burst: A phenomenon where coal and rock in mines suddenly fracture and release energy, often leading to the destruction of mine tunnels or working faces.
Bursting liability: Refers to the potential or tendency for rock or coal seams to experience shock ground pressure when subjected to external forces.
Working face: In coal mining, the location or area where coal mining operations are directly carried out, representing the forefront of coal production.
Immediate roof: In coal mining, the rock layer directly above the coal seam.
Mudstone: A fine-grained sedimentary rock, primarily composed of tiny mineral particles, typically smaller than 0.0625 mm in size. Mudstone is harder when dry and may become softer when wet.
Mining depth: The vertical distance from the surface to the operational face of the mine.
Coal seam: A layer in the underground sedimentary rock formation, primarily composed of coal and serving as the main source of coal resources.
Basic roof: In coal mining, the harder or more stable rock layer located directly above the immediate roof.
Siltstone: A fine-grained sedimentary rock, composed of mineral particles with sizes between those of mudstone and sandstone.
Immediate floor: In coal mining, the rock layer directly beneath the coal seam.
Main overlying roof: The principal rock layer located directly above the immediate roof, usually harder and more stable, playing a crucial role in the overall stability of the mine.
Sandstone: A medium to coarse-grained sedimentary rock, primarily made up of sand-sized mineral particles, known for its good porosity and permeability
Grey argillaceous rocks: Sedimentary rocks rich in clay minerals, giving the rock a muddy texture. These rocks are typically grey in color, varying in shades due to the presence of organic matter or iron.
Charcoal mudstone: A type of mudstone that contains a higher proportion of carbonaceous material, usually darker in color, ranging from grey-black to black.
Fine-siltstone: A fine-grained sedimentary rock, composed of mineral particles sized between mudstone and sandstone, generally ranging from 0.0039 to 0.0625 mm.
Burial depth: The vertical distance from the surface to the position of a rock layer or stratum.
