# Supplementary material for: Classification of coal bursting liability of some chinese coals using machine learning methods
Source: Sci Rep. 2024 Jun 18;14:14030. doi: 10.1038/s41598-024-61801-0 (PMC11189441; doi:10.1038/s41598-024-61801-0)
Supplement: Supplementary file 1 — Supplementary Information. [file 41598_2024_61801_MOESM1_ESM.docx]

**Sample database of coal bursting liability**

| No. | Sample source | *DT* (ms) | *W*_ET_ | *K*_E_ | *R*_C_ (MPa) | Engineering Guidance Level |
| --- | --- | --- | --- | --- | --- | --- |
| 1 | A Coal Mine | 461 | 2.23 | 1.45 | 7.3 | 2 |
| 2 | A Coal Mine | 537 | 2.08 | 2.38 | 7.79 | 2 |
| 3 | A Coal Mine | 674 | 2.23 | 2.46 | 8.66 | 2 |
| 4 | A Coal Mine | 409 | 2.16 | 1.39 | 8.16 | 2 |
| 5 | A Coal Mine | 2943 | 1.103 | 2.17 | 2.193 | 3 |
| 6 | Chengjiao Coal Mine | 306 | 5.91 | 2.48 | 8.86 | 2 |
| 7 | Qianqiu Coal Mine | 160.5 | 7.1 | 7.08 | 25.64 | 1 |
| 8 | Qianqiu Coal Mine | 102 | 2.67 | 2.26 | 13.31 | 2 |
| 9 | Pingmei No.13 Coal Mine | 22611 | 0.63 | 2.71 | 3.93 | 3 |
| 10 | Silou Coal Mine | 193 | 1.14 | 5.74 | 12.52 | 2 |
| 11 | Mayangcun Coal Mine | 3469 | 1.25 | 4.55 | 16.45 | 3 |
| 12 | Datong Mining Area | 5.7 | 9.1 | 1.14 | 2.1 | 2 |
| 13 | Datong Mining Area | 5 | 6.3 | 1.9 | 2.01 | 2 |
| 14 | Datong Mining Area | 33 | 5.2 | 69 | 2.84 | 1 |
| 15 | Datong Mining Area | 3.75 | 4.85 | 112 | 6.3 | 1 |
| 16 | Datong Mining Area | 7.25 | 4.8 | 96 | 5.8 | 1 |
| 17 | Datong Mining Area | 6.9 | 5.9 | 67 | 5.4 | 1 |
| 18 | Datong Mining Area | 12.8 | 6.5 | 123 | 5.1 | 1 |
| 19 | Datong Mining Area | 14.2 | 3.4 | 66 | 5.67 | 1 |
| 20 | Guangzheng Coal Mine | 9.3 | 5.1 | 31.2 | 5.7 | 1 |
| 21 | A Coal Mine | 170.6 | 10.27 | 1.912 | 24.892 | 1 |
| 22 | A Coal Mine | 13.8 | 4.1 | 112 | 3.23 | 1 |
| 23 | A Coal Mine | 140.2 | 2.245 | 8.808 | 16.224 | 1 |
| 24 | Taoshan Coal Mine | 50.35 | 6.71 | 4.52 | 11.78 | 2 |
| 25 | Hauting Coal Mine | 73 | 2.96 | 19.37 | 14.67 | 2 |
| 26 | Hauting Coal Mine | 60 | 2.93 | 13.51 | 14.32 | 2 |
| 27 | Yuejin Coal Mine | 351 | 2.63 | 1.64 | 12.98 | 2 |
| 28 | Chengshan Coal Mine | 255 | 3.4 | 3.7 | 11.15 | 2 |
| 29 | Dongtai Coal Mine | 212 | 4.34 | 0.88 | 7.31 | 2 |
| 30 | Guojiahe Coal Mine | 267 | 12.4 | 0.87 | 24.77 | 1 |
| 31 | Sanhejian Coal Mine | 54 | 19.63 | 1.29 | 17.25 | 1 |
| 32 | A Coal Mine | 362.8 | 6.02 | 1.34 | 8.61 | 2 |
| 33 | Baodian Coal Mine | 42 | 14.41 | 3.63 | 29.04 | 1 |
| 34 | Baodian Coal Mine | 42 | 21.9 | 5.35 | 26.63 | 1 |
| 35 | Xinzhouyao Coal Mine | 13.28 | 3.67 | 5.67 | 29.17 | 1 |
| 36 | A Coal Mine | 44.5 | 4.34 | 5.99 | 22.63 | 1 |
| 37 | A Coal Mine | 139.8 | 3.19 | 3.34 | 13.29 | 2 |
| 38 | Yuncheng Coal Mine | 316 | 8.1 | 1.4 | 16.7 | 1 |
| 39 | Chenjiagou Coal Mine | 68.3 | 12.69 | 6.73 | 14.57 | 1 |
| 40 | A Coal Mine | 5230 | 0.62 | 1.361 | 2.42 | 3 |
| 41 | Yanbei Coal Mine | 248.57 | 2.15 | 1.84 | 12.03 | 2 |
| 42 | Junde Coal Mine | 346 | 11.3 | 6.45 | 15.24 | 1 |
| 43 | Zhangji Coal Mine | 52.5 | 6.62 | 4.32 | 9.52 | 2 |
| 44 | Daanshan Coal Mine | 138.4 | 0.968 | 3.541 | 9.969 | 2 |
| 45 | Hauting Coal Mine | 45 | 12.3 | 12.57 | 18.77 | 1 |
| 46 | Hauting Coal Mine | 33 | 10.28 | 9.84 | 12.09 | 1 |
| 47 | Hauting Coal Mine | 82 | 4.78 | 10.18 | 13.9 | 2 |
| 48 | Hauting Coal Mine | 20 | 9.43 | 8.72 | 16.5 | 1 |
| 49 | Hauting Coal Mine | 105 | 7.71 | 4.21 | 13.89 | 2 |
| 50 | Baotailong Mining Area | 750.3 | 8.19 | 1.36 | 9.52 | 3 |
| 51 | Baotailong Mining Area | 421.8 | 22.67 | 1.39 | 17.4 | 1 |
| 52 | Xinxing Coal Mine | 41.2 | 11.91 | 11.767 | 5.489 | 1 |
| 53 | Yuncheng Coal Mine | 340 | 4.58 | 1.26 | 15.82 | 2 |
| 54 | Yuejin Coal Mine | 275 | 3.53 | 3.56 | 13.08 | 2 |
| 55 | A Coal Mine | 47 | 9.2 | 4.13 | 17.4 | 1 |
| 56 | A Coal Mine | 31.33 | 17.5 | 5.42 | 11.93 | 1 |
| 57 | Pingmei No.1 Coal Mine | 1149 | 3.39 | 3.45 | 14.38 | 2 |
| 58 | Yuejin Coal Mine | 65.89 | 10.42 | 6.3 | 28.6 | 1 |
| 59 | Tangshan Coal Mine | 306 | 1.63 | 2.06 | 9.93 | 2 |
| 60 | Taoshan Coal Mine | 54.82 | 4.67 | 4.93 | 15.99 | 2 |
| 61 | Pingdingshan Mining Area | 24 | 4 | 2.85 | 23.85 | 1 |
| 62 | Pingdingshan Mining Area | 301 | 3.84 | 2.47 | 10.2 | 2 |
| 63 | Pingdingshan Mining Area | 92 | 4.3 | 4.53 | 14.65 | 2 |
| 64 | Hongyang Mining Area | 252 | 3.11 | 1.93 | 6.51 | 2 |
| 65 | Chaoyang Coal Mine | 23.41 | 49 | 5.09 | 4.59 | 1 |
| 66 | Babao Coal Mine | 12333 | 0.96 | 1.14 | 4.56 | 3 |
| 67 | Zhaolou Coal Mine | 42 | 7.39 | 5.67 | 20.5 | 1 |
| 68 | Zhaolou Coal Mine | 46 | 8.12 | 10.63 | 25.9 | 1 |
| 69 | Hujiahe Coal Mine | 39.8 | 6.49 | 7.73 | 24.27 | 1 |
| 70 | Hujiahe Coal Mine | 34.4 | 4.45 | 12.57 | 24.35 | 1 |
| 71 | Chaoyang Coal Mine | 215.93 | 2.62 | 1.81 | 13.11 | 2 |
| 72 | Yaoqiao Coal Mine | 19 | 7.42 | 13.67 | 9.72 | 1 |
| 73 | Daoqing Coal Mine | 315.67 | 3.47 | 2.27 | 4.93 | 2 |
| 74 | Tendong Coal Mine | 43.2 | 14.6 | 11.8 | 34.06 | 1 |
| 75 | Chengshan Coal Mine | 284 | 3.96 | 1.84 | 10.5 | 2 |
| 76 | Yima Coal Mine | 256 | 3.96 | 2.31 | 16.59 | 2 |
| 77 | Gaole Coal Mine | 167 | 17.603 | 15.682 | 22.597 | 1 |
| 78 | Gaole Coal Mine | 69.2 | 12.522 | 35.723 | 33.907 | 1 |
| 79 | Nanshan Coal Mine | 2624 | 1.417 | 5.978 | 14.04 | 2 |
| 80 | Muchengjian Coal Mine | 30 | 10.9 | 14.8 | 29.3 | 1 |
| 81 | Taoshan Coal Mine | 391 | 6.286 | 1.4778 | 9.4 | 2 |
| 82 | Xinxing Coal Mine | 1173 | 11.91 | 11.767 | 7.85 | 1 |
| 83 | Jixian Coal Mine | 288 | 1.63 | 2.342 | 15.378 | 2 |
| 84 | Jixian Coal Mine | 239 | 1.4 | 2.029 | 11.138 | 2 |
| 85 | A Coal Mine | 423 | 5.332 | 2.632 | 27.28 | 2 |
| 86 | Zhujidong Coal Mine | 706.66 | 1.87 | 1.836 | 9.78 | 2 |
| 87 | Dongtan Coal Mine | 133 | 7.6 | 4.86 | 19.6 | 1 |
| 88 | Pingmei No.4 Coal Mine | 156 | 4.19 | 3.67 | 14.44 | 2 |
| 89 | Pingmei No.4 Coal Mine | 375 | 2.1 | 1.93 | 11.43 | 2 |
| 90 | Pingmei No.4 Coal Mine | 137 | 5.28 | 4.15 | 13.75 | 2 |
| 91 | Pingmei No.4 Coal Mine | 258 | 2.01 | 2.05 | 12.49 | 2 |
| 92 | Pingmei No.4 Coal Mine | 185 | 2.78 | 3.26 | 13.28 | 2 |
| 93 | Datai Coal Mine | 463.778 | 3.161 | 1.5 | 8.9 | 2 |
| 94 | Changyugou Coal Mine | 212.8 | 2.06 | 2.68 | 21.42 | 2 |
| 95 | Xinglu Coal Mine | 287 | 9.18 | 4.99 | 9.53 | 2 |
| 96 | Xinzhouyao Coal Mine | 15 | 3.37 | 5.248 | 30 | 1 |
| 97 | A Coal Mine | 48 | 8.06 | 9.4 | 10.58 | 1 |
| 98 | Gengcun Coal Mine | 90 | 2.5 | 2.6 | 9.51 | 2 |
| 99 | A Coal Mine | 47 | 9.07 | 40.17 | 72.45 | 1 |
| 100 | Chenman Coal Mine | 483 | 2.08 | 2.1 | 1.56 | 2 |
| 101 | A Coal Mine | 260 | 1.88 | 1.67 | 12.87 | 2 |
| 102 | A Coal Mine | 189 | 6.05 | 6.49 | 18.64 | 1 |
| 103 | Yuejin Coal Mine | 258 | 7.181 | 5.264 | 15.211 | 1 |
| 104 | Xinan Coal Mine | 324 | 3.769 | 1.545 | 10.946 | 2 |
| 105 | A Coal Mine | 345 | 3 | 2.79 | 9.7 | 2 |
| 106 | Zhaoguan Energy | 39 | 10.89 | 11.61 | 16.41 | 1 |
| 107 | Zhaoguan Energy | 68 | 3.44 | 17.89 | 12.55 | 2 |
| 108 | Zhaoguan Energy | 147 | 4.27 | 5.96 | 11.29 | 2 |
| 109 | Zhaoguan Energy | 31 | 9.13 | 8.49 | 10.87 | 1 |
| 110 | Zhaoguan Energy | 133 | 4.62 | 5.77 | 9.42 | 2 |
| 111 | Zhaoguan Energy | 72 | 4.09 | 9.47 | 11.59 | 2 |
| 112 | Zhaoguan Energy | 16 | 8.71 | 8.13 | 14.37 | 1 |
| 113 | Zhaoguan Energy | 94 | 7.19 | 4.69 | 11.55 | 2 |
| 114 | Zhaoguan Energy | 235 | 3.38 | 3.01 | 10.67 | 2 |
| 115 | Binchang Coal Mine | 72 | 13.35 | 6.2 | 19.37 | 1 |
| 116 | A Coal Mine | 49.667 | 8.472 | 9.921 | 29.886 | 1 |
| 117 | Anju Coal Mine | 161.4 | 3.382 | 2.253 | 13.79 | 2 |
| 118 | Jiangou Coal Mine | 285 | 1.91 | 2.02 | 10.1 | 2 |
| 119 | A Coal Mine | 3492 | 6.3 | 9.75 | 22.44 | 1 |
| 120 | A Coal Mine | 31325 | 0.2 | 1.37 | 2.04 | 3 |
| 121 | Gengcun Coal Mine | 254 | 3 | 1.94 | 7.31 | 2 |
| 122 | Bulianta Coal Mine | 49.667 | 8.472 | 65.92 | 31.661 | 1 |
| 123 | A Coal Mine | 357 | 4.6 | 2.9 | 11.5 | 2 |
| 124 | A Coal Mine | 494 | 1.37 | 2.88 | 10.81 | 2 |
| 125 | A Coal Mine | 415 | 4.38 | 1.13 | 8.2 | 2 |
| 126 | Datong Mining Area | 66.4 | 4 | 1.7 | 1.8 | 2 |
| 127 | Anju Coal Mine | 161.4 | 3.382 | 2.253 | 13.79 | 2 |
